# Oncological benefits of portal vein embolization for patients with hepatocellular carcinoma

**DOI:** 10.1002/ags3.12414

**Published:** 2020-12-13

**Authors:** Toru Beppu, Kensuke Yamamura, Hirohisa Okabe, Katsunori Imai, Hiromitsu Hayashi

**Affiliations:** ^1^ Department of Surgery Yamaga City Medical Center Kumamoto Japan; ^2^ Department of Gastroenterological Surgery Graduate School of Life Sciences Kumamoto University Kumamoto Japan

**Keywords:** hepatocellular carcinoma, oncological benefit, portal vein embolization, portal vein ligation

## Abstract

Portal vein embolization (PVE) for hepatocellular carcinoma (HCC) was first introduced in 1986 and has been continuously developed throughout the years. Basically, PVE has been applied to expand the indication of liver resection for HCC patients of insufficient future liver remnant. Importantly, PVE can result in tumor progression in both embolized and non‐embolized livers; however, long‐term survival after liver resection following PVE is at least not inferior compared with liver resection alone despite the smaller future liver remnant volume. Five‐year disease‐free survival and 5‐year overall survival were 17% to 49% and 12% to 53% in non‐PVE patients, and 21% to 78% and 44% to 72% in PVE patients, respectively. At present, it has proven that PVE has multiple oncological advantages for both surgical and nonsurgical treatments. PVE can also enhance the anticancer effects of transarterial chemoembolization and can avoid intraportal tumor cell dissemination. Additional interventional transarterial chemoembolization and hepatic vein embolization as well as surgical two‐stage hepatectomy and associated liver partition and portal vein ligation for staged hepatectomy can enhance the oncological benefit of PVE monotherapy. Taken together, PVE is an important treatment which we recommend for listing in the guidelines for HCC treatment strategies.

## Graphical abstract

Oncological benefits of portal vein embolization (PVE) for hepatocellular carcinoma were summarized. PVE can result in tumor progression in both embolized and non‐embolized livers; however, long‐term survival after liver resection following PVE is at least not inferior compared with liver resection alone despite the smaller future liver remnant volume. Further, PVE has multiple oncological advantages for both surgical and nonsurgical treatments.

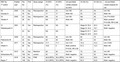

## 1 INTRODUCTION

Portal vein embolization (PVE) has been carried out for various hepatobiliary malignancies such as hepatocellular carcinoma (HCC).^1^, ^2^, ^3^, ^4^, ^5^, ^6^, ^7^, ^8^, ^9^ PVE for HCC was first introduced in 1986 by the Osaka City University group in Japan.^1^ PVE is mainly done to obtain a larger future liver remnant (FLR) to expand the safety zone of liver resection. Even for HCC patients with fibrous livers, liver resectability is increased after PVE without increasing morbidity and mortality.^3^, ^5^, ^7^ Previous studies have assessed liver resectability based on liver function and FLR volume using computed tomography (CT)‐volumetry.^8^, ^9^, ^10^, ^11^ Nowadays, functional liver volumetry using ^99m^Tc‐galactosyl human serum albumin scintigraphy single‐photon emission CT is used to assess partial liver function after PVE.^12^, ^13^, ^14^, ^15^, ^16^ PVE can provide a larger functional volume of the FLR as compared to those before PVE. Additionally, the functional volume ratio after PVE has been found to be significantly greater than that of the traditional volume ratio of the remnant liver.^16^

In regard to patients with HCC, PVE can provide not only an increased remnant liver volume but also an enhanced effect of transarterial treatments^17^, ^18^, ^19^, ^20^, ^21^, ^22^, ^23^ and the prevention of transportal metastases to non‐embolized areas.^24^ PVE has a lower direct therapeutic effect on HCC, thus it can cause tumor progression while waiting for liver regeneration.^25^, ^26^, ^27^, ^28^, ^29^ Previous papers have confirmed comparable disease‐free survival (DFS) and overall survival (OS) rates for HCC patients undergoing major hepatectomy with or without PVE.^3^, ^5^, ^30^, ^31^, ^32^ In contrast, two papers have demonstrated a better DFS or OS in patients that had received PVE as compared to the patients who did not.^33^, ^34^ Whether PVE might show a better influence on recurrence or long‐term prognosis after major hepatic resection remains controversial.

There have been numerous review articles about PVE; however, articles specific for HCC remain limited and all of them mainly discussed the PVE procedure and liver regeneration effect.^35^, ^36^, ^37^ In this review, we will summarize the role of PVE for HCC with special attention to oncological effects.

## 2 INFLUENCE OF PVE ON TUMOR PROGRESSION

Several studies have suggested that tumor progression can occur after PVE in both embolized and non‐embolized livers; however, data remain inconclusive.^25^, ^26^, ^27^, ^28^, ^29^ Tumor progression after PVE has been reported to be influenced by the following factors: (a) malignant potential of the primary tumor, (b) alterations of hepatic blood supply to the tumor, (c) acceleration of inflammatory cytokines and growth factors, and (d) an enhanced cellular host response.^25^, ^26^, ^38^ Unilateral reduction of portal blood flow after PVE causes a compensatory increase in hepatic artery blood perfusion (hepatic arterial buffer response). As HCC tumors are mainly fed by arterial blood supply, PVE can potentiate local tumor growth.^39^ Using a rat portal vein ligation (PVL) model, it was found that hepatocyte growth factor (HGF) mRNA levels increased to a detectable level 6 to 24 hours after the operation in non‐ligated lobes, but was only slightly elevated in ligated atrophic lobes.^40^, ^41^ We had also reported a clinical investigation that showed a transient increase in the serum HGF level after PVE.^42^ Some HCC cells are known to express c‐met receptors. The autocrine and paracrine activation of the HGF‐c‐met pathway plays an important role in the progression of HCC.^43^

Tumor growth after PVE was observed in embolized and non‐embolized livers in patients with colorectal liver metastases^24^, ^44^, ^45^ For HCC, Hayashi et al^25^ investigated liver tumor growth in an embolized liver after PVE. They observed that the rate of tumor growth after PVE increased 4‐fold that before PVE (0.59 cm^3^/day to 2.37 cm^3^/day). There were no significant correlations between tumor growth in an embolized liver and the regeneration of non‐embolized liver parenchyma.

In the clinical setting, tumor growth in the non‐embolized residual liver is more important because tumors in the embolized liver can be removed by planned hepatic resection. Patients with bilateral multiple malignant liver tumors that require a two‐stage hepatectomy (TSH) may be the best candidates for PVE because contralateral tumors can be resected during the first operation.^46^

One important issue is the frequency of patient dropout after PVE during the waiting period of planned hepatectomy. Cancellation of the hepatic resection may occur because of insufficient liver hypertrophy, deterioration of liver function, and tumor progression. In regard to tumor progression, 4.2% to 11.1% of HCC patients were reported to have cancelled the planned hepatic resection after PVE.^35^, ^47^, ^48^ Furthermore, PVE in combination with major hepatectomy may be avoided for HCC patients with impaired liver function categorized as liver damage B by the Liver Cancer Study Group of Japan.^49^, ^50^ This may be due to the higher dropout rate after PVE and inadequate long‐term outcomes after hepatectomy.

## 3 EFFECTS OF PREOPERATIVE PVE ON LONG‐TERM OUTCOME

The DFS and OS for HCC after major hepatectomy with or without PVE are summarized in Table 1.^3^, ^5^, ^30^, ^31^, ^32^, ^33^, ^34^ Majority of the previous studies had suggested that PVE showed no obvious differences for recurrence and long‐term prognosis.^3^, ^5^, ^30^, ^31^, ^32^ 5Y‐DFS and 5Y‐OS were 17% to 49% and 12% to 53% in non‐PVE patients, and 21% to 78% and 44% to 72% in PVE patients, respectively. However, Tanaka et al^33^ had reported better OS rates in HCC patients with PVE only in patients whose 15‐min indocyanine retention rate was over 13% as compared to patients without PVE. Additionally, we have previously demonstrated that preoperative PVE was one of the independent predictors by multivariate analysis for favorable DFS in patients with HCC that required major hepatectomy.^34^ Table 1 contains data limited to patients with HCC who underwent major hepatectomy after PVE; therefore, some selection bias may exist. An intention‐to‐treat analysis is strongly recommended to compare all patients who received PVE and those with initially resectable HCC who did not receive PVE. However, from a different perspective, we have to consider that PVE is always applied only in patients with HCC and insufficient FLR and, therefore, there are obvious background differences that cannot be corrected in the PVE and non‐PVE groups. In fact, our multicenter study^5^ showed a significantly smaller initial %FLR in the PVE group compared with the non‐PVE group (40% vs 52%) even after propensity score‐matching (PSM) analysis. After PVE, the %FLR was identical in the two groups (50% vs 52%).

**Table 1:** Long‐term outcome after hepatectomy for hepatocellular carcinoma patients with or without portal vein embolization

First author Ref no.	Publish year	No. pts	PVE	Study design	3Y‐DFS (%)	5Y‐DFS (%)	Uni‐ and multivariate analysis for DFS	3Y‐OS (%)	5Y‐OS (%)	Uni‐ and multi‐variate analysis for OS
Azoulay^30^	2000	10	Yes	Retrospective	64	21	Uni: NS Multi: NA	67	44	Uni: NS Multi: NA
		19	No		17	17		53	53	
Tanaka^33^	2000	33	Yes	Retrospective	NA	33	Uni: NS Multi: NA	NA	50	Uni: *P* = .0024 Multi: positive^#^ *P* = .028, RR 0.346
		38	No		NA	20		NA	25	
Wakabayashi^31^	2001	26	Yes	Retrospective	NA	NA	NA	Stage III: 50.4 Stage IV: 44.4	40.3 16.7	Uni: NA Multi: negative
		43	No		NA	NA		Stage III: 61.7 Stage IV: 22.5	46.3 22.5	
Palavecino^3^	2009	21	Yes	Retrospective	56	56	Uni: NS Multi: NA	82	72	Uni: NS Multi: NA
		33	No		49	49		63	54	
Okabe^34^	2011	19	Yes	Retrospective	77.7	77.7	Uni: 0.01 Multi: positive *P* = .02, HR 3.59	72.3	72.3	Uni: 0.049 Multi: NA
		36	No		19.6	0		57.2	12.3	
Siriwardana^32^	2012	34	Yes	Matched control	29	26	Uni: NS Multi: negative	73	63	Uni: NS Multi: negative
		102	No		42	42		62	52	
Beppu^5^	2016	148	Yes	PSM	46.8 (RFS)	36.4 (RFS)	Uni: NS Multi: negative	65.5	58.6	Uni: NS Multi: negative
		148	No		42.3 (RFS)	35.3 (RFS)		63.3	52.8	

Abbreviations: DFS, disease‐free survival; HR, hazard ratio; NA., not available; NS, not significant; OS, overall survival; Positive^#^, positive for limited patients with 15‐min indocyanine green retention rate of at least 13%; PSM, propensity matching study; PVE, portal vein embolization; Ref no., reference number; RFS, recurrence‐free survival; RR, risk ratio.

**Figure 1:**
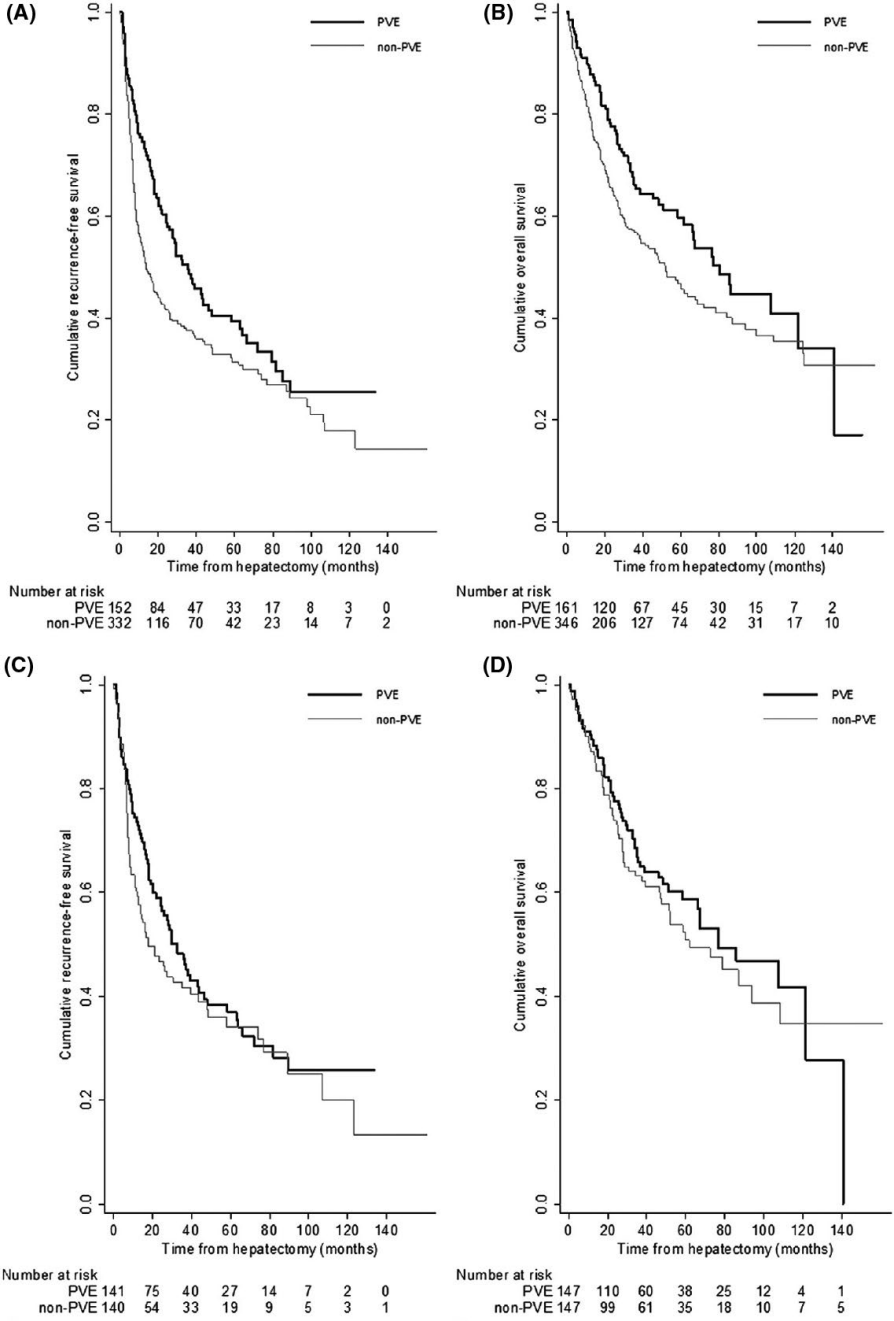
Cumulative survival curves in the portal vein embolization (PVE) group and in the non‐PVE group. (Reproduced with permission from Beppu et al^5^) (A) Recurrence‐free survival (RFS) and (B) Overall survival (OS) in the overall cohort before propensity score‐matching (PSM). (C) RFS and (D) OS in the PSM cohort. In the overall cohort, RFS and OS in the PVE group were significantly greater than those of the non‐PVE group (*P* < .005 for RFS and *P* < .037 for OS). In the PSM cohort, RFS and OS were not significantly different in the two groups (*P* = .281 for RFS and *P* = .519 for OS)

To resolve these clinical questions, we conducted a multicenter study using PSM analysis for patients with HCC (≥5 cm) that underwent PVE followed by right‐sided hemi‐hepatectomy.^5^ In the overall cohort of patients with or without PVE before PSM, RFS and OS in the PVE group were significantly greater than those in the non‐PVE group (*P* < .005 for RFS and *P* < .037 for OS) (Figure 1A,B); however, the application of PVE was not an independent prognostic factor for RFS and OS by multivariate analysis. In contrast, in the PSM cohort, patients treated with PVE showed at least a non‐inferior long‐term prognosis as compared to patients undergoing upfront hepatectomy despite the smaller FLR (Figure 1C,D). Furthermore, 10 random PSM analyses (Table 2) demonstrated median *P* values (ranges) of .153 (.048‐.334) for RFS and .209 (.019‐.519) for OS in the PVE and in the non‐PVE group. The smallest *P* values were significant for both; therefore, preoperative PVE may have a potential to provide better RFS and OS. The reasons for better long‐term outcomes in PVF patients included the following: (a) suitable patient selection: new lesions in the remnant liver or extrahepatic lesions may be detected during the waiting time for liver resection, (b) support of postoperative remnant liver function after hepatic resection,^34^ (c) and prevention of portal dissemination of tumor cells caused by liver manipulation during liver resection.^51^ Interestingly, extrahepatic recurrences were more frequent in the non‐PVE group. It is unclear whether PVE could affect tumor cell migration into the hepatic vein. In order to resolve this issue, further molecular studies for disseminated tumor cells in the liver vessels are required.^52^, ^53^ Oppositely, one paper had reported higher rates of postoperative distant metastasis in the PVE group.^31^

**Table 2:** Ten random trials for propensity matching

Trial	*P*‐value for recurrence‐free survival	*P*‐value for overall survival
1	.281	.519
2	.160	.076
3	.260	.235
4	.048	.053
5	.052	.019
6	.179	.183
7	.146	.240
8	.065	.291
9	.081	.063
10	.334	.293

Note

Reproduced with permission from Beppu et al.^5^

Recently, a unique paper had shown that disease progression after PVE did not affect long‐term outcomes for patients with HCC if the planned hepatectomy was completed.^47^ Disease progression was defined as increases in tumor size, number, or markers. Disease progression was observed in 14.0% to 47.4% of the patients; however, this was not an independent prognostic factor by multivariate analysis. In contrast, for patients with colorectal liver metastases, it has been reported that PVE could induce tumor progression and provide poorer long‐term survival rates for patients with tumor progression.^26^, ^38^, ^45^

## 4 CLINICAL BENEFITS OF PVE IN NONSURGICAL THERAPY

The indication of PVE for HCC patients who required portal vein occlusion other than liver regeneration for major hepatectomy is summarized (Table 3). There were a few reports demonstrating total or subtotal pathological necrosis of HCC after PVE monotherapy.^54^, ^55^, ^56^ These results were not predicted because HCC was fed by a predominant arterial flow.^38^ This may be due to the fact that well‐differentiated HCC could instead be mainly fed by portal flow.

**Table 3:** Indication of portal vein occlusion for HCC patients other than liver regeneration

1. PVE for portal vein derived HCC
2. Additional PVE on TACE monotherapy
3. PVE to avoid intraportal dissemination of ablation therapy
4. PVL to prevent extension of PVTT
5. Transient portal vein occlusion for HCC patients with AP shunt

Abbreviations: AP shunt, arterioportal shunt; HCC, hepatocellular carcinoma; PVE, portal vein embolization; PVL, portal vein ligation; PVTT, portal vein tumor thrombosis; TACE, transarterial chemoembolization.

Intrahepatic metastases of HCC have been known to usually occur in the portal vein; therefore, PVE may inhibit transportal dissemination of the tumor cells into the non‐embolized area. We have also reported that PVE combined with TACE for HCC patients can prevent intrahepatic recurrence to the non‐PVE area and improve long‐term outcomes.^24^ All patients had unresectable HCC tumors in one lobe, which were treated with repeated TACE alone or TACE + PVE during the same period. With the exception of gender, the background factors did not differ between patients in the two groups. Of all the patients undergoing TACE + PVE, approximately half were initially scheduled for surgery but were still considered to have unresectable HCC even after PVE, while the other half received PVE mainly to reduce intrahepatic metastases to the contralateral lobe. Repeated TACE without PVE was performed in patients for whom PVE was technically difficult and for those who were unable to provide consent for PVE. Overall intrahepatic recurrence rates were comparable; however, the recurrence rate limited to the non‐portal‐embolized area was significantly lower in the TACE + PVE group as compared to that in the TACE group (58.8% vs 81.8% at 3‐years; *P* = .026). OS rate was significantly higher in the TACE + PVE group than in the TACE group (38.2% vs 9% at 5‐years; *P* = .045). Even in patients in whom additional major hepatectomy is impossible, sufficient benefits may be received from performing additional PVE on TACE.

Portal vein tumor thrombosis (PVTT) can easily grow in the contralateral portal system. Ablation therapy for HCC patients adjacent to the large Glissonean pedicle can provide intrahepatic dissemination via the portal vein.^57^ Depending on the method used to prevent migration of HCC cells or spread of PVTT, PVE has also been conducted.^25^, ^58^, ^59^, ^60^, ^61^ For patients with PVTT, PVL is preferable because PVE is not tight enough to suppress extension of PVTT.^61^

The arterioportal shunt (AP shunt) in HCC patients can sometimes prevent achieving TACE, which can deteriorate liver function and may be a poor prognostic factor.^62^, ^63^ Percutaneous transhepatic transient portal vein occlusion has been reported as a beneficial tool to complete sufficient TACE for HCC patients with an AP shunt.^64^, ^65^ Transient portal vein occlusion can provide significantly better therapeutic effects, including an improved tumor response and long‐term survival rates as compared to conventional shunt embolization that uses coils and/or gelatin‐sponge particles.

## 5 ENHANCEMENT OF ONCOLOGICAL EFFECT OF PVE

### 5.1 Additional TACE on PVE

Using a rabbit VX2 liver tumor model, the TACE + PVE group clearly showed the strongest suppressive effect on tumor growth and induced the highest level of tumor cell apoptosis among the TACE, PVE, and Sham operation groups.^66^ TACE solo therapy has been known to obtain a higher tumor necrosis effect in about half of HCC patients after TACE.^67^ In the clinical setting, the basic concept of additional TACE on PVE is to increase the FLR hypertrophy rate, and there have already been reports of excellent additional effects on liver regeneration.^17^, ^18^, ^19^, ^20^, ^21^, ^22^, ^23^ For patients with insufficient volume of FLR immediately after PVE, avoiding tumor progression by TACE is necessary during the waiting period.^34^

There have been four papers comparing long‐term outcomes between HCC patients treated with TACE plus PVE versus PVE alone followed by major hepatectomy (Table 4).^18^, ^19^, ^21^, ^23^ The 5‐year DFS and 5‐year OS were 35%‐61% and 43%‐84% in the PVE + TACE group, and 0%‐38% and 20%‐58% in the PVE‐alone group, respectively. By univariate analysis, the PVE + TACE group provided better RFS and OS compared with the PVE‐alone group. However, the data were inconclusive because there were no multivariate analyses, PSM studies as well as RCT. Recently, intent‐to‐treat analysis data were published investigating sequential TACE plus PVE (n = 27) versus PVE alone (n = 28) before major hepatectomy for patients with large HCC (≥5 cm).^23^ Baseline characteristics were equivalent in the two groups. The number of dropout patients for liver resection were only two (9%) in the TACE + PVE group and nine (32%) in the PVE‐alone group. OS was significantly better in the former as compared to the latter (3‐year OS of 60% vs 20%; *P* = .01).

**Table 4:** Long‐term outcome after hepatectomy for hepatocellular carcinoma patients with PVE + TACE versus PVE alone

1st author Ref no.	Publish year	No. pts	Preoperative therapy	Study design	3Y‐RFS (%)	5Y‐RFS (%)	Uni‐ and multivariate analysis for DFS	3Y‐OS (%)	5Y‐OS (%)	Uni‐ and multivariate analysis for OS
Ogata^18^	2006	18	PVE + TACE	Retrospective	37	37	Uni: *P* = .041 Multi: NA	54	43	Uni: NS Multi: NA
		18	PVE alone		19	19		31	31	
Yoo^19^	2011	71	PVE + TACE	Retrospective	70	61	Uni: *P* = .028 Multi: NA	83	72	Uni: *P* = .001 Multi: NA
		64	PVE alone		51	38		73	56	
Choi^21^	2015	27	PVE + TACE	Retrospective	NA	NA	NA	83.4	83.4	Uni: *P* = .047 Multi: NA
		13	PVE alone		NA	NA		76.9	57.7	
Terasawa^23^	2020	21	PVE + TACE	Retrospective	28 (PFS)	NA	Uni: *P* = .03 Multi: NA	55	NA	Uni: NS Multi: NA
		19	PVE alone		0	NA		28	NA	
		27	PVE + TACE	Retrospective ITT analysis	35 (PFS)	NA	Uni: *P* < .01 Multi: NA	60	NA	Uni: *P* = .01 Multi: NA
		28	PVE alone		0	NA		20	NA	

Abbreviations: ITT, intent‐to‐treat; NA, not available; NS, not significant; OS, overall survival; PFS, progression‐free survival; PVE, portal vein embolization; Ref no., reference number; RFS, recurrence‐free survival; TACE, transarterial chemoembolization.

Sequential TACE and PVE have been carried out as an order of TACE followed by PVE with an interval of 2 or 3 weeks.^17^, ^18^, ^19^, ^20^, ^21^, ^22^, ^23^ In contrast, our group performed with an inverted order.^34^ The reasons for the “PVE‐first approach” included: (a) the extent of liver regeneration depended on the interval period between PVE and liver resection, and (b) PVE was required to achieve complete obliteration, so regulation of the procedure was difficult. In the PVE‐first approach, delicate TACE can be applied with minimal arterial obstruction of the surrounding liver tissue in the portal embolized liver.

### 5.2 Additional hepatic vein embolization on PVE

Recently, staged and simultaneous preoperative portal and hepatic vein embolization (biembolization) have been introduced and described to create higher liver hypertrophy than PVE alone before major liver resection.^68^, ^69^ However, this approach involves a prolonged waiting period and thus further increases the risk of tumor progression. Unfortunately, oncological effects of biembolization for HCC have not been fully investigated.^68^ It is noteworthy that additional HVE on PVE might be able to decrease both intra‐ and extrahepatic metastases.

### 5.3 Advances in operative procedure using PVE

Two‐stage hepatectomy has been developed for bilateral liver tumors. The first step includes tumor enucleation of the FLR followed by PVE or PVL, while the second step involves major hepatectomy.^70^, ^71^, ^72^ Associated liver partition and PVL for staged hepatectomy (ALPPS) is a novel operative procedure consisting of two steps: (a) PVE or PVL and liver transection with or without tumor enucleation from the residual liver, and (b) major hepatectomy.^70^, ^71^, ^72^

ALPPS technique has been described to obtain an increased volume of PVE and a decrease in dropout rates.^70^, ^71^, ^72^ ALPPS could be a feasible technique only in selected patients with HCC and cirrhosis.^71^, ^72^ As long as it is performed in an experienced center, it may be used over PVE or could be used as a rescue technique in the case of PVE failure. In hepatitis‐related HCC patients, ALPPS can provide a higher resection rate and comparable short‐ and long‐term oncological outcomes as compared to PVE followed by major hepatectomy.^71^ The 3‐ and 5‐year DFS and OS rates were 34.9%, and 25.0%, and 41.8%, and 40.7%, respectively (*P* = .267), and 60.2% and 46.8%, and 73.5%, and 64.1%, respectively (*P* = .234), in the ALPPS and PVE groups. Recently, several types of modified ALPPS procedure including laparoscopic partial ALPPS have been developed mainly to decrease morbidity and mortality.^73^, ^74^

For patients with colorectal liver metastases, ALPPS can provide greater liver hypertrophy in a shorter period as compared to TSH; however, in some papers, early recurrence and poor OS is indicated.^75^, ^76^ Excessive production of various inflammatory cytokines and growth due to rapid liver regeneration is thought to be one of the reasons. Thus, further studies comparing ALPPS and TSH for HCC are strongly required.

In conclusion, PVE has been developed mainly to achieve hypertrophy of the non‐embolized liver. We would like to emphasize that PVE followed by major hepatectomy for initially unresectable HCC can at least result in non‐inferior long‐term survival compared with initially resectable HCC without PVE. It has now spread worldwide and can provide multiple oncological advantages for both surgical and nonsurgical treatments and is recommended for inclusion in the guidelines for HCC treatment strategies.^77^

## DISCLOSURE

Conflicts of Interest: Authors declare no conflicts of interest for this article.
